# Roles of miR-10a-5p and miR-103a-3p, Regulators of BDNF Expression in Follicular Fluid, in the Outcomes of IVF-ET

**DOI:** 10.3389/fendo.2021.637384

**Published:** 2021-05-12

**Authors:** Qiyao Zhang, Jinfeng Su, Wei Kong, Zhou Fang, Yan Li, Ziqiang Huang, Ji Wen, Yue Wang

**Affiliations:** ^1^ Reproductive Medical Center, The Second Hospital Affiliated to Shandong University of Traditional Chinese Medicine, Jinan, China; ^2^ Institute of Basic Medicine, Shandong Provincial Hospital Affiliated to Shandong First Medical University, Jinan, China

**Keywords:** oocyte maturation, microRNA (miR), IVF-ET, follicular fluid (FF), brain-derived neurotrophic factor (BDNF), blastocyte

## Abstract

Brain-derived neurotrophic factor (BDNF), a member of the neurotrophin family, plays critical roles in the physiological process of oocyte mature and IVF outcomes of patients with infertility. However, the regulation of BDNF expression in the microenvironment surrounding the oocyte is still unknown. We initially predicted some microRNA (miRNA) candidates targeting *bdnf* with a series of bioinformatics analysis tools to determine the underlying regulatory mechanisms of BDNF, particularly the effect of miRNAs on BDNF expression. Then, we assessed whether the expression of these 14 selected miRNAs was negatively associated with BDNF expression in follicular fluid (FF) samples obtained from mature (>18 mm) or immature (<15 mm) follicles. Finally, we used the candidate miRNAs, miR-103a-3p and miR-10a-5p, to further investigate the relationship between their expression in FF and the outcomes of infertile patients undergoing IVF–ET treatment. The results of the bioinformatics analysis revealed 14 miRNAs that might directly regulate BDNF expression and might have a close relationship with oocyte development. BDNF was expressed at significantly lower levels in FF from immature follicles than in FF from mature follicles, and only the expression of miR-103a-3p and miR-10a-5p was negatively correlated with BDNF expression in FF. Moreover, in another cohort of 106 infertile women undergoing IVF-ET treatment, miR-103a-3p or miR-10a-5p expression predicted the developmental status of the corresponding oocytes in which high expression of miR-103a-3p or miR-10a-5p resulted in a poor quality of embryo on days 3 and 5 during the IVF-ET treatment. In conclusion, our study is the first to show that miR-103a-3p or miR-10a-5p negatively affects the maturation of oocytes by regulating the expression of BDNF in human FF. Additionally, the expression levels of miR-103a-3p or miR-10a-5p in FF may predict the outcomes of IVF, which are helpful for improving embryo selection and consequently the IVF success rate in the clinic.

## Introduction

The adult ovary, a vital dynamic organ, has the potential to regularly develop and produce mature oocytes capable of fertilization from the ovarian reserve of numerous immature follicles (1). The process of folliculogenesis is tightly regulated by both systemic hormonal signaling and the local microenvironment of the ovary (2). Any changes in these factors caused by aging, polycystic ovarian syndrome (PCOS) or ovarian endometriosis may block the development of oocytes and lead to infertility.

Brain-derived neurotrophic factor (BDNF), an important member of the neurotrophin family, exerts well-known trophic effects on neuronal survival, neurogenesis and synaptic plasticity (3). Additionally, BDNF, together with its high-affinity tyrosine kinase type B (TrkB) receptor, are expressed in gonadotrophin-releasing hormone (GnRH) neurons, endocrine glands, and adult mammalian ovaries (4); furthermore, the concentrations of BDNF in peripheral blood and ovarian follicular fluid (FF) exhibit dynamic changes during the menstrual cycle of normally cycling fertile women or in the process of controlled ovarian stimulation for *in vitro* fertilization (IVF) (5–7), implying a crucial role for BDNF in the regulation of female reproductive physiology. Although inconsistencies in the predictive value of circulating BDNF in natural fertility and IVF success have been reported (4), the local BDNF expression levels in the ovary are positively associated with ovarian follicle formation and oocyte maturation and survival (4, 8).

The expression of the *bdnf* gene in the ovary is regulated by multiple factors, including gonadotrophins, adrenal and gonadal steroids, and epigenetic mechanisms (4). As one of the important mechanisms of epigenetic regulation, microRNAs (miRNAs) negatively modulate the expression of their target genes by binding the 3 ‘UTR region, and miRNAs are very common in mammals. Based on the results of *in vitro* studies, miR-10a-5p, 101 and 204 contribute to the mechanism regulating BDNF expression in cervical and ovarian cancer cells (9–11). As shown in the study by Peng et al., miR-10b suppresses goat granulosa cell proliferation by targeting BDNF expression (12). However, currently, the knowledge of whether or which miRNAs regulate BDNF expression in the human ovary during *in vitro* fertilization and embryo transfer (IVF–ET) is still limited.

We initially predicted some miRNA candidates targeting *bdnf* with a series of bioinformatics analysis tools to provide clearly answers to the questions described above. Then, we assessed whether the expression of these miRNAs was negatively associated with BDNF expression in FF derived from mature (>18 mm) or immature (<15 mm) follicles. Finally, we used the candidate miRNAs to further investigate whether these miRNAs represent potential biomarkers for predicting the outcomes of IVF–ET.

## Materials and Methods

### Patients Recruitment

This prospective observational study recruited women with IVF-ET treatment at Reproductive Medical Center of the Second Hospital Affiliated to Shandong University of Traditional Chinese Medicine (Jinan, Shandong, China) between May 2018 and March 2019. Patients with polycystic ovary syndrome (PCOS), endometriosis, premature ovarian failure, unexplained infertility and whose partner had any male infertile factors were excluded from the study. Clinical characteristics of patients were collected from electronic patient charts. This study was performed in accordance with the guidelines in the Declaration of Helsinki and was approved by the Medical Ethics Committee of the Second Hospital Affiliated to Shandong University of Traditional Chinese Medicine. All patients signed the informed consent.

### Measurement of Follicular-Fluid BDNF Levels

Levels of BDNF were determined by using the commercially available BDNF Emax Immunoassay System (BDNF Emaxw ImmunoAssay System, Promega, USA) according to our previous Study (13). Briefly, 96-well plates were coated with anti-BDNF monoclonal antibody overnight at 4°C, and then were blocked with blocking buffer. After that, the FF and standards were added and incubated for 2 h at room temperature. The reporter antibody (Anti-human BDNF polyclonal antibody), anti-IgY–horseradish peroxidase conjugate and the chromogenic substrate were given subsequently. 1 N hydrochloric acid was used to stop of the reaction. The absorbency was measured at 450 nm by using a microplate reader (Infinite F50, Tecan, Austria). All samples were assayed in duplicate.

### Collection of Biological Samples

Blood samples were obtained between 7:30 a.m. and 8:00 a.m. on day 3 of the menstrual cycle. Follicular fluid was collected during routine egg retrieval from infertile women with a tubal obstruction who were undergoing controlled ovarian stimulation in preparation for IVF. The FF sample was collected through the transvaginal ultrasound guided puncture of a specific size of follicle according to the experimental design. Aspirates were obtained from the first follicle from either side in an effort to obtain clear FF. After removing the oocyte, the fluid was processed by centrifugation at 300 g for 10 min, and the clear supernatant was stored at -80°C until use.

### Follicle Stimulation Protocol and Oocyte Retrieval

The GnRH agonist long protocol was used in all patients, as described in a previous study (13). Briefly, GnRH agonist, decapeptyl (0.1 mg/ampoule, Ferring, Germany), was administered in the midluteal phase of the previous cycle until the day of triggering. After menstrual bleeding and a satisfactory pituitary desensitization was achieved (serum 17β-estradiol level was <180 pmol/l), recombinant FSH, Gonal F (75 U/ampoule, Serono Ltd., Switzerland), began to be used. The dose of Gonal F was adjusted according to the size of follicles and serum 17β-estradiol (E2) concentrations. HCG (10000 IU) (Lizhu Ltd., Guangdong, China) was administered when at least two follicles reached a mean diameter of 18 mm. Thirty-six hours later, transvaginal follicular aspiration was performed for oocyte retrieval. The oocytes were considered mature if a polar body appeared by 4–6 h after insemination. The fertilization of the oocyte was determined by the appearance of two pronuclei. Good quality embryos were defined as embryos that developed from normal fertilized eggs with no fragmentation or no more than one-third fragmentation, without multinucleated cells, and the presence of three to five blastomeres at 48 h after egg retrieval and at least seven blastomeres by 72 h. The top quality of blastocyst on day 5 is defined as fully expanded or hatching, with a prominent inner cell mass (ICM) and cohesive trophectoderm (TE).

### Extraction and Analysis of miRNAs

**Figure 1:**
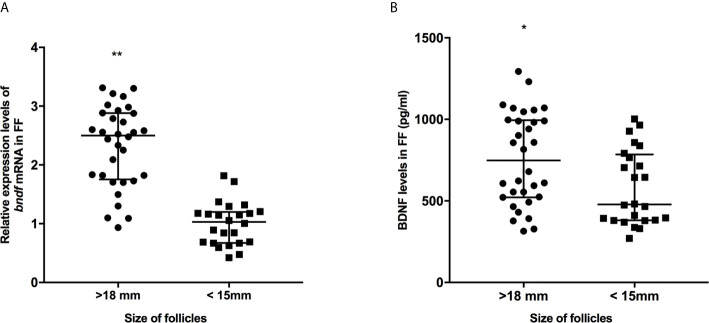
The expression levels of *bdnf* **(A)** and BDNF proteins **(B)** in FF from mature (>18 mm) and immature (<15 mm) follicles. *P < 0.05; **P < 0.01. Data are presented as the mean ± standard deviation. Student’s t-test or Mann-Whitney tests were performed to determine the differences between two groups based on the normality of the distribution of data.

**Figure 2:**
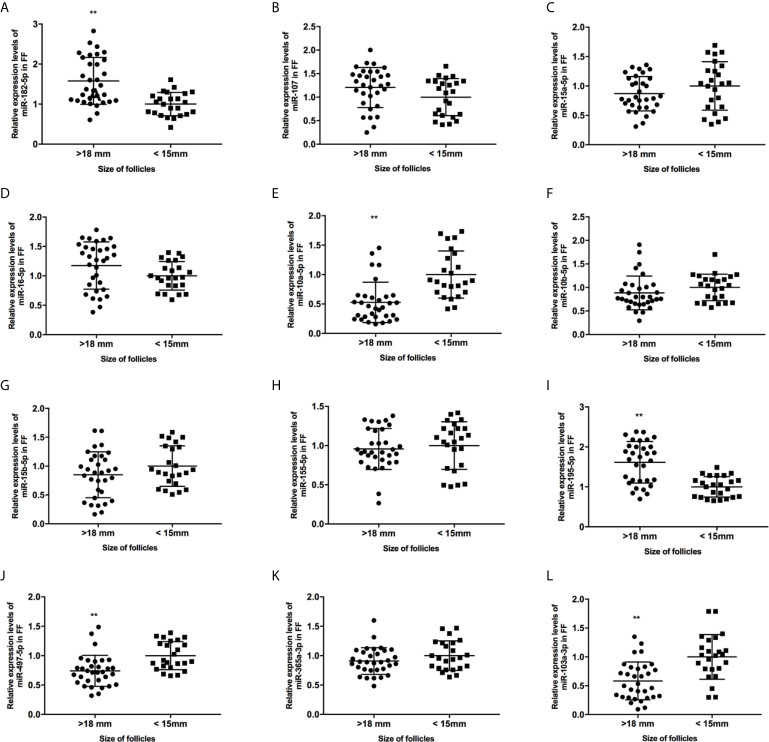
The expression levels of candidate miRNAs in FF from mature (>18 mm) and immature (<15 mm) follicles **(A–L)**. **, P < 0.01. Data are presented as the mean ± standard deviation. Student’s t-test or Mann-Whitney tests were performed to determine the differences between two groups based on the normality of the distribution of data.

RNA was extracted and purified using the miRNeasy Kit (QIAGEN, Hilden, Germany). Briefly, 200 μl of FF supernatant from each patient were mixed thoroughly with 1000 μl of QIAzol^®^ Lysis Reagent (QIAGEN) in centrifuge tubes. After a 5 min incubation at 24°C, 200 μl of chloroform were added to the mixture, which was vortexed vigorously for 15 s. The RNA pellet was collected by centrifugation at 12,000×g for 15 min at 4°C. The upper aqueous phase was collected in a new tube with 1.5 volumes of 100% ethanol. The mixture was pipetted up and down several times. Then, the sample was transferred to an RNeasy MinElute spin column in a 2 ml collection tube and centrifuged at ≥ 8000×g for 15 s at room temperature. The RNA pellet was further sequentially washed with Buffer RWT, Buffer RPE and 80% ethanol. The lid of the spin column was opened and centrifuged at full speed for 5 min to dry the membrane. Finally, the pellet was dissolved in 30 μl of RNase-free H_2_O. The miRNAs were quantified with the miScript PCR Systems (QIAGEN) according to the manufacturer’s protocol. The expression of miRNAs was normalized to the U6 snRNA. The relative expression levels of miRNAs were calculated using the formula 2^−ΔΔCt^ in **Figures 1**, **2** and using the formula 2^−ΔCt^ ×1000 in **Tables 1**, **2**.

**Table 1:** Correlation between miR-103a-3p or miR-10a-5p expression and Clinical and biochemical characteristics of patient with IVF-ET treatment.

Variables		N	miR-103a-3p	P-value	miR-10a-5p	P-value
		(total = 106)	Median (Q1–Q3)		Median (Q1–Q3)	
Maternal age (years)	<37	77	8.00 (5.08-10.84)	0.913	1.89 (0.75-3.04)	0.962
	≥37	29	8.83 (3.65-12.11)		1.62 (0.77-3.08)	
BMI (kg/m2)	<18.5	4	8.74 (5.49-10.90)	0.488	2.27 (0.90-3.57)	0.777
	18.5≤BMI<25	70	8.42 (4.96-11.45)		1.80 (0.64-3.00)	
	≥25	32	5.73 (3.11-11.98)		1.94 (1.14-3.15)	
FSH (IU/l)	<10	89	7.56 (4.21-10.54)	0.079	1.50 (0.75-2.71)	0.003
	≥10	17	11.7 (6.42-14.97)		3.93 (1.97-4.31)	
LH (IU/l)	≤4	58	7.55 (3.82-9.39)	0.03	1.08 (0.55-2.18)	<0.001
	<4	48	9.66 (5.56-13.22)		2.93 (1.41-3.75)	
E2 (pg/ml)	≤45	52	8.34 (4.15-11.97)	0.48	1.37 (0.57-2.91)	0.118
	<45	54	7.62 (4.47-11.28)		2.12 (1.07-3.16)	
T (pg/ml)	≤30	102	7.92 (4.47-11.32)	0.459	1.66 (0.73-2.92)	0.02
	<30	4	8.90 (4.47-40.71)		3.52 (2.77-4.11)	
AMH (ng/ml)	<2	31	9.11 (4.54-12.28)	0.256	2.14 (1.09-3.29)	0.196
	≥2	75	7.70 (4.27-11.06)		1.62 (0.57-2.97)	
P4 (ng/ml)	≤4	96	7.85 (4.34-11.19)	0.271	1.66 (0.70-2.95)	0.205
	<4	10	9.51 (6.68-13.80)		2.74 (1.09-3.62)	
Diagnosis	Primary infertility	66	8.30 (4.79-11.69)	0.772	2.07 (1.08-3.93)	0.128
	Secondary infertility	40	7.62 (3.79-11.28)		1.44 (0.54-2.70)	

Median (Q1–Q3), median (25th - 75th percentiles). BMI, body mass index; FSH, follicle-stimulating hormone; LH, luteinizing hormone; E2,17β-estradiol; T, testosterone; AMH, anti-Müllerian hormone; AFC, antral follicle count. Values of miRNAs are calculated by 2 -ΔCt ×1000, ΔCT = Raw Ct (miRNA) -Raw Ct (U6).

**Table 2:** Correlation between miR-103a-3p or miR-10a-5p expression in FF and their corresponding oocyte developmental statues during IVF treatment.

ART outcomes		N	miR-103a-3p	P-value	miR-10a-5p	P-value
		(total = 106)	Median (Q1–Q3)		Median (Q1–Q3)	
Mature oocytes	Yes	102	8.14 (4.24-11.25)	0.531	1.75 (0.76-3.01)	0.697
	No	4	10.53 (5.38-17.51)		2.43 (0.66-3.57)	
2PN	Yes	80	7.50 (3.80-10.59)	0.062	1.49 (0.64-2.76)	0.002
	No	26	10.73 (6.60-12.92)		3.02 (1.11-3.83)	
Top quality embryos on day 3	Yes	52	7.46 (3.80-9.12)	0.029	1.35 (0.51-2.32)	<0.001
	No	54	9.66 (5.71-13.12)		2.69 (1.17-3.61)	
Top quality blastocyst on day5	Yes	39	7.56 (4.54-9.21)	0.047	1.50 (0.63-2.66)	0.019
	No	67	8.67 (4.27-12.66)		2.20 (0.91-3.53)	

Median (Q1–Q3), median (25th - 75th percentiles) Values of miRNAs are calculated by 2 -ΔCt ×1000, ΔCT = Raw Ct (miRNA) - Raw Ct (U6).

### Statistical Analysis

Continuous parametric data are presented as the mean ± standard deviations (SD) or median (25^th^ - 75^th^ percentiles). Student’s t-test or Mann-Whitney tests were performed to determine the differences between two groups based on the normality of the distribution of data. One-way ANOVAs were used to analyze the differences among three groups. We calculated the Pearson coefficient to assess the correlation between the expression of *bdnf* and miRNAs in FF. All statistical analysis were performed using SPSS software (version 19.0; SPSS Inc., Chicago, IL, USA). P values < 0.05 were considered statistically significant.

## Results

### Prediction of miRNAs Targeting BDNF to Regulate Its Expression

According to previous reports, the concentration of BDNF in FF fluctuates with the menstrual cycle or IVF process, and is involved in the maturation of oocytes (5, 6). If *bdnf* expression is regulated by miRNAs during these processes, we want to know which miRNA is the most likely participant. Therefore, in the first step, we used an open-source analysis platform, the Encyclopedia of RNA Interactomes (ENCORI, http://starbase.sysu.edu.cn), to predict the miRNAs that might bind to and downregulate *bdnf* (14). Here, we used a set of stringent parameters to refine our results: CLIP evidence (>=2), program number (>=3, and one must be identified using Targetscan). We filtered out 21 mRNAs (**Table 3**). Then, we further assessed the functional effects of these miRNAs on biological processes and pathways using DIANA-miRPath v3.0 (http://snf-515788.vm.okeanos.grnet.gr). Some pathways related to oocyte development were significantly enriched, including the cell cycle (hsa04110), oocyte meiosis (hsa04114), neurotrophin signaling pathway (hsa04722), and progesterone-mediated oocyte maturation (hsa04914). Fourteen miRNAs were involved in all these pathways, suggesting that these miRNAs might regulate the expression of *bdnf* and be involved in the maturation of oocytes.

**Table 3:** The list of primarily screened miRNAs that interact with BDNF by ENCORI.

miRNAid	miRNAname
MIMAT0000068	hsa-miR-15a-5p
MIMAT0000069	hsa-miR-16-5p
MIMAT0000101	hsa-miR-103a-3p
MIMAT0000104	hsa-miR-107
MIMAT0000253	hsa-miR-10a-5p
MIMAT0000254	hsa-miR-10b-5p
MIMAT0000259	hsa-miR-182-5p
MIMAT0000416	hsa-miR-1-3p
MIMAT0000417	hsa-miR-15b-5p
MIMAT0000461	hsa-miR-195-5p
MIMAT0000462	hsa-miR-206
MIMAT0000646	hsa-miR-155-5p
MIMAT0000710	hsa-miR-365a-3p
MIMAT0000736	hsa-miR-381-3p
MIMAT0001341	hsa-miR-424-5p
MIMAT0002817	hsa-miR-495-3p
MIMAT0002820	hsa-miR-497-5p
MIMAT0003281	hsa-miR-613
MIMAT0004903	hsa-miR-300
MIMAT0022479	hsa-miR-5688
MIMAT0022834	hsa-miR-365b-3

### miR-10a-5p and miR-103a-3p Negatively Correlate With BDNF Expression in FF

**Figure 3:**
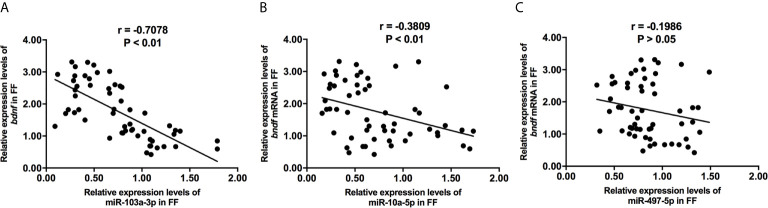
The correlations of miR-103a-3p **(A)**, miR-10a-5p **(B)** and miR-497-5p **(C)** expression with bdnf expression in FF from mature (>18 mm) and immature (<15 mm) follicles. Pearson coefficient was used to assess the correlation.

We collected 32 (Age, 28.3 ± 4.3; BMI (body mass index), 24.5 ± 3.9) and 24 (Age, 27.1 ± 3.7; BMI, 23.9 ± 3.2) patients’ FF samples from mature (>18 mm) or immature (<15 mm) follicles during IVF treatment, respectively, and detected the expression of BDNF and miRNAs in these samples to verify the roles of the miRNAs predicted above in regulating BDNF expression and oocyte development. It showed no obvious differences in age and BMI values between the two groups (Both P >0.05). Higher levels of both the BDNF mRNA and protein were detected in FF from mature follicles than in FF from the immature follicles (**Figures 1A, B**, P < 0.01 and P < 0.05). Among the 14 miRNAs, the expression of miR-497-5p, miR-10a-5p and miR-103a-3p was decreased (**Figure 2**, P < 0.01), and the expression of the remaining miRNAs either displayed no changes, increased significantly or was undetected in the FF from mature follicles compared with the FF from immature follicles. Moreover, the expression of both miR-103a-3p and miR-10a-5p displayed more negative correlations with BDNF mRNA levels in FF (**Figure 3**, both P < 0.01). Based on these results, miR-103a-3p and miR-10a-5p might be the key miRNAs that inhibited the expression of BDNF in FF and negatively regulated the maturation of oocytes.

### The Relationship Between miR-103a-3p or miR-10a-5p Expression and the Clinical Characteristics of Patients Undergoing IVF-ET

Considering the association between the expression of miRNAs and BDNF in FF, we further investigated whether the expression of miR-103a-3p or miR-10a-5p predicted the outcomes of IVF-ET. For this experiment, an additional cohort of 106 infertile women with IVF-ET were analyzed. The expression of miR-103a-3p did not correlate with most clinical parameters, including maternal age, body mass index (BMI), state of infertility, and the basal levels of most hormones, with the exception of the level of luteinizing hormone (LH) (**Table 1**, P < 0.05). The expression of miR-10a-5p showed an increasing trend in patients with higher levels of follicle stimulating hormone (FSH, **Table 1**, P < 0.01), LH (**Table 1**, P < 0.01) or testosterone (T, **Table 1**, P < 0.05). These results suggested a positive correlation between our selected miRNAs and the basal levels of some hormones.

### The Relationship Between miR-103a-3p or miR-10a-5p Expression and the Outcomes of Patients Undergoing IVF-ET

Next, we analyzed the possible roles of miR-103a-3p or miR-10a-5p in the outcomes of IVF-ET. We observed a moderate increase in miR-103a-3p expression in the subgroups in which oocytes did not develop into high-quality embryos on day 3 or day 5 (both P < 0.05). Moreover, an obvious increase in miR-10a-5p expression was detected in the subgroups without the formation of 2 pronuclei (P < 0.01) and subgroups in which oocytes did not develop into high-quality embryos on day 3 (P < 0.01) or day 5 (P < 0.05). Thus, high expression of miR-103a-3p or miR-10a-5p in FF might predict a relatively poor outcome of embryonic development during IVF-ET treatment.

## Discussion

In this study, considering the important effects of BDNF on IVF outcomes, we initially screened some miRNAs that might regulate the expression of BDNF in the ovary. Among the 14 candidates, miR-103a-3p and miR-10a-5p expression were negatively correlated with the expression of *bdnf* in FF and might be involved in the maturation of oocytes in follicles. We recruited 106 patients undergoing IVF treatment to explore the relationship between the expression of miR-103a-3p or miR-10a-5p in FF with the developmental outcomes of corresponding oocytes and to further confirm this hypothesis. High expression of miR-103a-3p or miR-10a-5p in FF might predict a relatively poor outcome of embryonic development on day 3 and day 5 during IVF-ET treatment.

BDNF is mainly secreted from granular cells. BDNF in the FF plays an important role in the maturation of oocytes, fertilization, and early embryonic development, and this protein shows varying degrees of correlation with IVF outcomes in patients with different etiologies of infertility (4, 5, 8, 15). Clarification of the mechanisms regulating the transcription of BDNF in the ovary will be helpful for improving the outcomes of infertility treatments. In the present study, we identified miR-103a-3p and miR-10a-5p as two critical candidates that negatively regulate the expression of *bdnf* in FF. Notably, miR-103a-3p and miR-10a-5p are not only expressed in normal ovarian tissue but also in ovarian carcinoma tissues (16–18). The main effects of miR-10a-5p were on granulosa cell proliferation and apoptosis in previous studies (18–20). However, the physiological and pathological roles of miR-103a-3p in female reproduction remain obscure. To our knowledge, this report is the first to show that miR-103a-3p and miR-10a-5p expressed in the FF are involved in the development of the corresponding oocytes and better predict the outcomes of IVF in humans. Although previous findings and bioinformatics analysis all showed that miR-103a-3p and miR-10a-5p could regulate the *bdnf* expression through directly binding its 3 ‘UTR region (17, 21), the indirect effects of the two miRNAs on BDNF are not excluded. It is showed that some downstream targets of miR-103a-3p and miR-10a-5p, such as *htt, pten, arnt, rora* interact with BDNF either (22–25). Therefore, we suggest that the effects of miR-103a-3p and miR-10a-5p on the outcomes of IVF are probably achieved by regulating BDNF expression directly and indirectly.

Interestingly, high expression of miR-103a-3p or miR-10a-5p predicted the poor quality of cleavage-stage (day 3) and blastocyst-stage (day 5) embryos in our study. In recent years, an increasing trend of performing blastocyst-stage embryo transfer (BT) has been noted (26, 27). However, in this process, an increased incidence of transfer cancellations, extra time and labor costs, and a lower number of cryopreserved embryos have increased concerns related to the use of BT. Therefore, the establishment of an effective method to predict the development of blastocyst-stage embryos would be of high practical interest for IVF labs. A morphological assessment of embryos at specific developmental stages using time-lapse techniques is one useful method for selecting good blastocyst-stage embryos; however, expensive equipment and lower levels of predictability and accuracy are still shortcomings of this technique. A fast, convenient, and non-invasive approach to estimate the quality of blastocyst-stage embryos on day 5 in advance is needed. According to Canosa, et al., reduced levels of Cx43 expression in the cumulus cells, coupled with increased expression of the BMP-15 mRNA, are considered markers of a higher probability of blastocyst development on day 5 (28). Alfaidy, et al. identified PROK1 expression levels in individual FF samples as a predictor the *in vitro* fertilization (IVF) outcomes (29). Moreover, the expression of some non-coding RNAs, such as miR-451, in FF samples was positively correlated with the quality of blastocyst-stage embryos obtained from patients with endometriosis (30). In the present study, the expression levels of miR-103a-3p or miR-10a-5p in FF were closely related to the development of embryos, and thus they might be effective predictors for choosing a suitable IVF treatment strategy in advance.

In this study, patients with higher basal levels of follicle stimulating hormone (FSH) or luteinizing hormone (LH) displayed higher miR-103a-3p and miR-10a-5p expression in FF. In humans, changes in the expression of the BDNF mRNA or protein in reproductive tissues are mediated by complex processes that are similar to those in the central nervous system and regulated by neural activity, gonadotrophins, adrenal and gonadal steroids (4). As shown in previous studies, the expression of BDNF transcripts in granulosa cells increased 3-4 fold after treatment with the combination of FSH and hCG (31). Similarly, LH and hCG induced a robust increase in BDNF expression in cumulus granulosa cell (32). Because miR-103a-3p and miR-10a-5p may negatively regulate the expression of *bdnf*, we speculate that the synchronized changes in miR-103a-3p or miR-10a-5p expression along with basal FSH/LH levels might maintain the ovarian BDNF level in the appropriate range. Certainly, this hypothesis still need more evidence to verify in the following.

In conclusion, our study is the first to show that miR-103a-3p and miR-10a-5p negatively affect the maturation of oocytes by regulating the expression of BDNF in human FF. Additionally, the expression levels of miR-103a-3p and miR-10a-5p in FF may implicitly determine the quality of the embryo on days 3 and 5, which might be helpful for improving embryo selection and consequently the IVF success rate in the clinic. Besides, it should be noted that several limitations exist in our study. First, the relationship between miRNA and BDNF expression was detected in a relative small samples, and it is necessary to increase sample numbers for further validation in multi-centers in the future. Second, as an observational study, detailed interaction mechanisms and binding site of miRNA and BDNF were not considered in this study, in the next, more *in vitro* studies should be done to clarify above questions.

## Data Availability Statement

The original contributions presented in the study are included in the article/supplementary material. Further inquiries can be directed to the corresponding authors.

## Ethics Statement

The studies involving human participants were reviewed and approved by Medical Ethics Committee of the Second Hospital Affiliated to Shandong University of Traditional Chinese Medicine. The patients/participants provided their written informed consent to participate in this study.

## Author Contributions

YW and JW designed the study. QZ, JS, ZF, WK, and ZH performed all the experiments and analyzed the data. YL and JW recruited the patients. YW wrote the manuscript. All authors contributed to the article and approved the submitted version.

## Funding

This study was supported by National Natural Science Foundation of China (81771557), Academic Promotion Programme of Shandong First Medical University (2019QL016) and The Innovation Project of Shandong Academy of Medical Sciences.

## Conflict of Interest

The authors declare that the research was conducted in the absence of any commercial or financial relationships that could be construed as a potential conflict of interest.

The reviewer ZS declared a shared affiliation with some of the authors to the handling editor at time of review.
